# Imaging topological polar structures in marginally twisted 2D semiconductors

**DOI:** 10.1126/sciadv.aed8555

**Published:** 2026-09-11

**Authors:** Thi-Hai-Yen Vu, Daniel Bennett, Gayani Nadeera Pallewela, Johnathon Maniatis, Joshua Edwards, Md Hemayet Uddin, Kaijian Xing, Pablo Resendiz-Vazquez, Seng Huat Lee, Zhiqiang Mao, Jack B. Muir, Linnan Jia, Jeffrey A. Davis, Kenji Watanabe, Takashi Taniguchi, Shaffique Adam, Pankaj Sharma, Michael S. Fuhrer, Mark T. Edmonds

**Affiliations:** ^1^ School of Physics and Astronomy, Monash University, Clayton VIC 3800, Australia.; ^2^ John A. Paulson School of Engineering and Applied Sciences, Harvard University, Cambridge, Massachusetts 02138, USA.; ^3^ School of Electrical and Electronic Engineering, Nanyang Technological University Singapore, 50 Nanyang Avenue, 639798, Singapore.; ^4^ Centre for Advanced 2D Materials, National University of Singapore, 6 Science Drive 2, Singapore 117546.; ^5^ College of Science and Engineering, Flinders Microscopy and Microanalysis, Flinders University, Adelaide, South Australia 5042 Australia.; ^6^ Flinders Institute for Nanoscale Science and Technology, Flinders University, Adelaide, South Australia, 5042, Australia.; ^7^ Melbourne Centre for Nanofabrication, Victorian Node of the Australian National Fabrication Facility, Clayton 3168, VIC, Australia.; ^8^ Department of Physics, Pennsylvania State University, University Park, PA 16802, USA.; ^9^ 2D Crystal Consortium, Materials Research Institute, Pennsylvania State University, University Park, PA 16802, USA.; ^10^ Optical Sciences Centre, Swinburne University of Technology, Hawthorn, 3122, Victoria, Australia.; ^11^ ARC Centre of Excellence in Future Low-Energy Electronics Technologies, Swinburne University of Technology, Hawthorn, 3122, Victoria, Australia.; ^12^ Research Center for Electronic and Optical Materials, National Institute for Materials Science, 1-1 Namiki, Tsukuba 305-0044, Japan.; ^13^ Research Center for Materials Nanoarchitectonics, National Institute for Materials Science, 1-1 Namiki, Tsukuba 305-0044, Japan.; ^14^ Department of Material Science and Engineering, National University of Singapore, 9 Engineering Drive 1, 117575, Singapore.; ^15^ Department of Physics, Washington University in St. Louis, St. Louis, Missouri 63130, United States.; ^16^ ARC Centre of Excellence in Future Low Energy Electronics Technologies, UNSW Sydney, NSW, 2052, Australia.; ^17^ ARC Centre of Excellence in Future Low-Energy Electronics Technology, Monash University, Clayton, 3800, Victoria, Australia.; ^18^ ANFF-VIC Technology Fellow, Melbourne Centre for Nanofabrication, Victorian Node of the Australian National Fabrication Facility, Clayton, VIC 3168, Australia.

## Abstract

Moiré superlattices formed in van der Waals heterostructures by twisting, lattice mismatch and strain present an opportunity for creating metamaterials with unique properties absent in the individual layers. Ferroelectricity for example, arises from broken inversion symmetry in twisted and strained bilayers of 2D semiconductors with stacking domains of alternating out-of-plane polarization. However, the individual contributions of twist and strain to the formation of topological polar nanostructures remain unclear and experimentally challenging to resolve. Inversion symmetry breaking is predicted to generate in-plane polarization along the domain walls, forming topologically non-trivial Bloch-type merons (half-skyrmions) in twisted systems and Néel-type merons in strained systems. Here we utilize angle-resolved vector piezoresponse force microscopy to spatially resolve polarization components and topological polar nanostructures in marginally twisted bilayer WSe_2_, providing experimental evidence of topologically non-trivial meron/antimeron structures. This approach can be used to distinguish Bloch-type, Néel-type and hybrid structures, allowing us to quantify the separate contributions of strain and twist in a moiré superlattice, opening pathways for exploring twist-induced topology in engineered nano-devices.

## INTRODUCTION

**Fig. 1.**
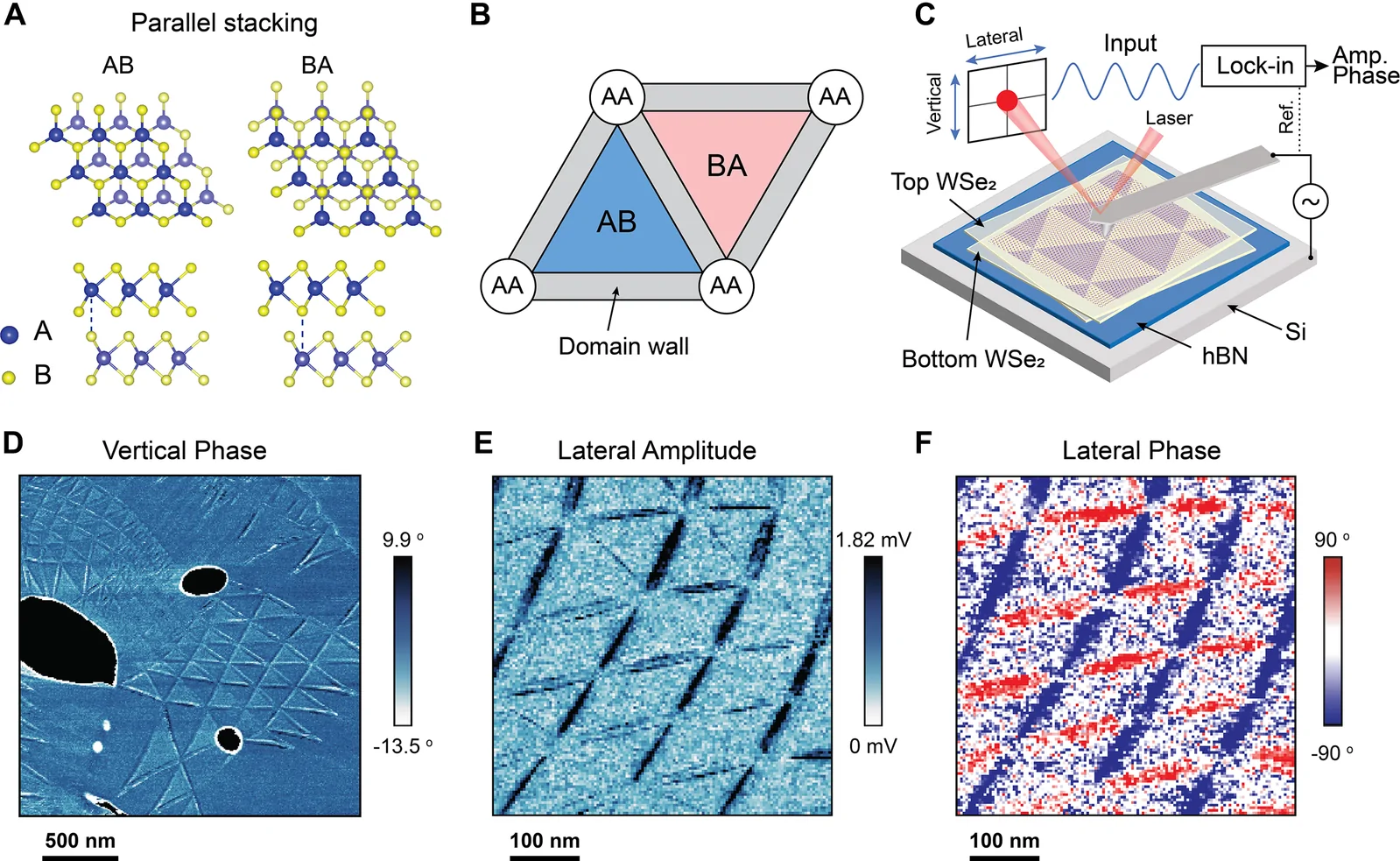
Piezoresponse force microscopy (PFM) visualization of marginally twisted bilayer TMDs. (**A**) Illustration of bilayer AB_2_ in the parallel stacking, where A is a transition metal (Mo, W) and B is a chalcogen (S, Se). The energetically favourable AB and BA stackings are shown, where the A and B atoms in neighbouring layers are vertically aligned. (**B**) Schematic depicting a moiré superlattice with AB (blue), BA (red), AA (white) stacking regions and domain walls (grey). (**C**) Schematic of PFM on a moiré pattern formed by marginally twisted bilayer WSe_2_. (**D**) Large-area (2.2 μm × 2.2 μm) vertical phase PFM image. (**E** and **F**) Lateral amplitude and phase PFM measurements taken within the same twisted bilayer region, respectively.

Stacking two-dimensional (2D) van der Waals (vdW) materials to form heterostructures has the potential to create new physical properties and functionalities. By twisting or straining layers with respect to one another, forming a moiré superlattice, a wide array of emergent properties has been realized such as superconductivity (*1*), correlated phases (*2*, *3*), magnetism (*4*) and even fractional Chern insulator states (*5*). The polar and electromechanical properties of moiré materials have also proven to be remarkably rich, where vdW systems with bipartite lattices lacking AB sublattice symmetry become ferroelectric by altering the relative stacking between the layers in order to break centrosymmetry (*6*, *7*) This is demonstrated for transition metal dichalcogenides in Fig. 1A (*8*): where the metals and chalcogens in neighbouring layers are vertically aligned (AB and BA stackings), the mirror plane between the layers is broken, resulting in an out-of-plane polarization via an interlayer electronic charge transfer. The AB and BA stackings have equal and opposite polarization as they are related by a mirror operation. An applied electric field can cause a relative sliding between the layers (vdW sliding) when the potential across the film is larger than the energy barrier between the AB and BA stackings, thus accessing bi-stable ground states, resulting in ferroelectricity (*7*). For marginally twisted bilayers, a network of AB and BA stacking domains form, separated by domain walls (DWs) as depicted in Fig. 1B. For TMDs with small twists, the stacking domains can be identified as a regular network of moiré polar domains (MPDs) (*9*). These MPDs can grow and shrink in response to an applied field (*10*, *11*) which is mediated by DW bending and motion. Under experimental conditions, due to domain wall pinning, the MPDs deform irreversibly, resulting in a switchable remnant polarization when the electric field is applied and removed (*12*–*15*).

Recently, it was proposed that the MPDs also have a spatially varying in-plane polarization component (*16*, *17*). Although the DW stacking, where locally there is a relative shift of half a unit cell long diagonal between the layers, does not possess a mirror symmetry, it is invariant under a mirror operation plus a non-symmorphic translation of half a unit cell diagonal, preventing any out-of-plane polarization. An in-plane polarization is not prevented by symmetry however, and the DW stacking has an in-plane polarization which is parallel to the relative shift between the layers. Thus, the MPDs in twisted and strained bilayers exhibit winding, transitioning from in-plane along the DWs to out-of-plane exactly at the AB/BA domain centers. In homobilayers, the winding in each MPD, including both the domain interiors and domain walls, is topologically nontrivial, with winding numbers of ±½, and the domain structure forms a regular network of merons and antimerons (half-skyrmions and half-antiskyrmions) (*16*). For twisted bilayers, the in-plane polarization circulates around the AB/BA domain centers, and the merons are of Bloch type, whereas for strained bilayers, i.e. one with a small lattice mismatch between the layers, the in-plane polarization flows into and out of the domain centers, and the merons are of Néel type. The meron topological texture that exhibits out-of-plane vectors at the core region and gradually changes to in-plane vectors has been found in both ferromagnetic and ferroelectric materials (*18*–*20*). Topological magnetic structures have applications in high density data storage due to their stability, as well as in logic gates (*21*). Topological polar structures, typically observed in oxide perovskite nanostructures (*22*), are thought to be advantageous for ultrafast energy storage, with phonon frequencies typically in THz range (*23*).

While very appealing both for fundamental physics in terms of investigating the connection between twist and topology and potential applications in nano-engineered devices that enable on-demand creation and manipulation of polar topological objects, the topological nature of the MPDs has been difficult to experimentally verify, primarily due to two factors. First, as the polarization in vdW materials is purely electronic, the in-plane component cannot be measured using the same techniques which are employed to measure the out-of-plane polarization, such as Kelvin probe force microscopy (*7*), resistance measurements (*13*) and electron microscopy (*15*). The in-plane polarization could in principle be measured from the lateral deflection from a piezoresponse force microscopy (PFM) tip, although the second issue is that in systems with small twist angles (large moiré periods), typically 1–2 degrees, notable lattice relaxation occurs (*24*, *25*), leading to sharp domain structures, with the in-plane polarization confined to the narrow domain walls, with widths of order 1 nm. Resolving the in-plane polarization would thus either require a very fine resolution, or larger domain walls, such as those in systems with very small twist angles (very large moiré periods), <0.5°. A nonzero phase has been measured along the domain walls in bilayer systems (*26*), primarily graphene, which is ordinarily nonpolar, although this was attributed to flexoelectricity due to the large strain gradient across narrow walls. Recently, several studies have reported experimental observations of in-plane polarization and topological polar textures in twisted van der Waals bilayers (*27*–*31*). While these works have established the existence of non-trivial polarization textures emerging in moiré materials, each has proposed differing interpretations of the observed topological textures and their underlying polarization configurations. A systematic approach combining direct measurements of the in-plane polarization vector field with theoretical modelling would provide a route to quantitatively distinguishing between Bloch-type, Néel-type and hybrid polar structures, and thus, establishing a consistent interpretation of topological polarization textures in moiré materials.

Here we report the observation of both out-of-plane and in-plane polarization in four marginally twisted WSe_2_ bilayer samples all with twist angle around 0.1° using PFM measurements at room temperature. By performing angle-resolved PFM measurements, we directly resolve the in-plane polarization, and we show that each MPD exhibits a clear winding confirming the existence of a topologically nontrivial meron-antimeron network in twisted bilayers. This is in excellent agreement with density functional theory (DFT) and molecular dynamics (MD) calculations predicting in-plane polarization is narrowly confined to the domain walls separating the polar domains. We identify a network of Bloch-type merons and antimerons in twisted bilayers, characterized by polarization parallel to domain walls. In regions with twist and strain, we detect hybrid merons and antimerons, exhibiting both Bloch and Néel characteristics, with polarization components parallel and perpendicular to domain walls. Our technique thus differentiates between heterostructures formed by twist versus lattice mismatch and can quantify the contributions of each in a moiré superlattice, which is not possible with out-of-plane polarization measurements alone.

## RESULTS

We performed non-invasive high-resolution piezoresponse force microscopy measurements to investigate the polar nanostructures and properties of marginally twisted WSe_2_. As shown optically in fig. S1A and schematically in Fig. 1C we fabricated four separate nearly 0° twisted bilayer WSe_2_ devices using the dry transfer technique (for details on sample fabrication, see Methods). Using second harmonic generation (see fig. S1B) the twist angle near 0° was confirmed prior to PFM imaging. The exact twist angle in the four twisted bilayer WSe_2_ devices ranging from 0.03 to 0.13° was determined using the moiré periodicity extracted directly from spatially resolved PFM measurements. Figure 1D shows a large area (2.2 μm × 2.2 μm) vertical PFM phase image taken in the twisted bilayer region of one of the WSe_2_ devices. In agreement with previous experimental studies (*14*, *15*), the vertical PFM phase map, which is related to the magnitude of the out-of-plane polarization and thus the piezoelectric coefficient (Fig. 1D) shows a clear triangular pattern, with AB and BA domains having opposite piezoelectric contrast. Evidence of the opposite out-of-plane polarizations which are relatively uniform across the domains and consistent with DFT and MD calculations shown in fig. S2, F and G and previous results (*14*, *15*). However, the periodicity of the triangular patterns varies as the local twist angle and/or layer-dependent strain changes due to wrinkles and bubbles that are observed in the corresponding AFM topography image of the same region (fig. S2, A and B). The enhanced vertical PFM phase signal near the DWs (as shown in fig. S2E) is due to large strain gradients which result in a polar response through flexoelectricity (*26*), which couples to piezoelectric stresses, resulting in two oppositely contrasting strong lines at the DWs. The in-plane flexoelectric response however is negligible (*26*), see Section S3 for discussion.

By capturing vertical and lateral deflections of the reflected laser on the quadrant photodiode detector independently, PFM can be used to detect not just the out-of-plane but also in-plane polarization responses. Lateral deflection results from in-plane torsion perpendicular to the cantilever axis. On the other hand, vertical deflection includes surface deformation resulting from both out-of-plane polarization and in-plane polarization components parallel to the cantilever axis (see fig. S2C). By performing lateral PFM amplitude and phase measurements in bilayer regions with twist angle (∼0.13°) we observe that the lateral response in Fig. 1E is confined solely to the narrow domain walls, with minimal response observed in the interior of AB and BA domains. This distinctly suggests the presence of in-plane polarization localized to the domain walls. The corresponding lateral phase image in Fig. 1F which indicates the polarization direction, shows there is a clear 180° phase switch between the alternating domain walls (histogram analysis of the phase switch is found in fig. S3).

**Fig. 2.**
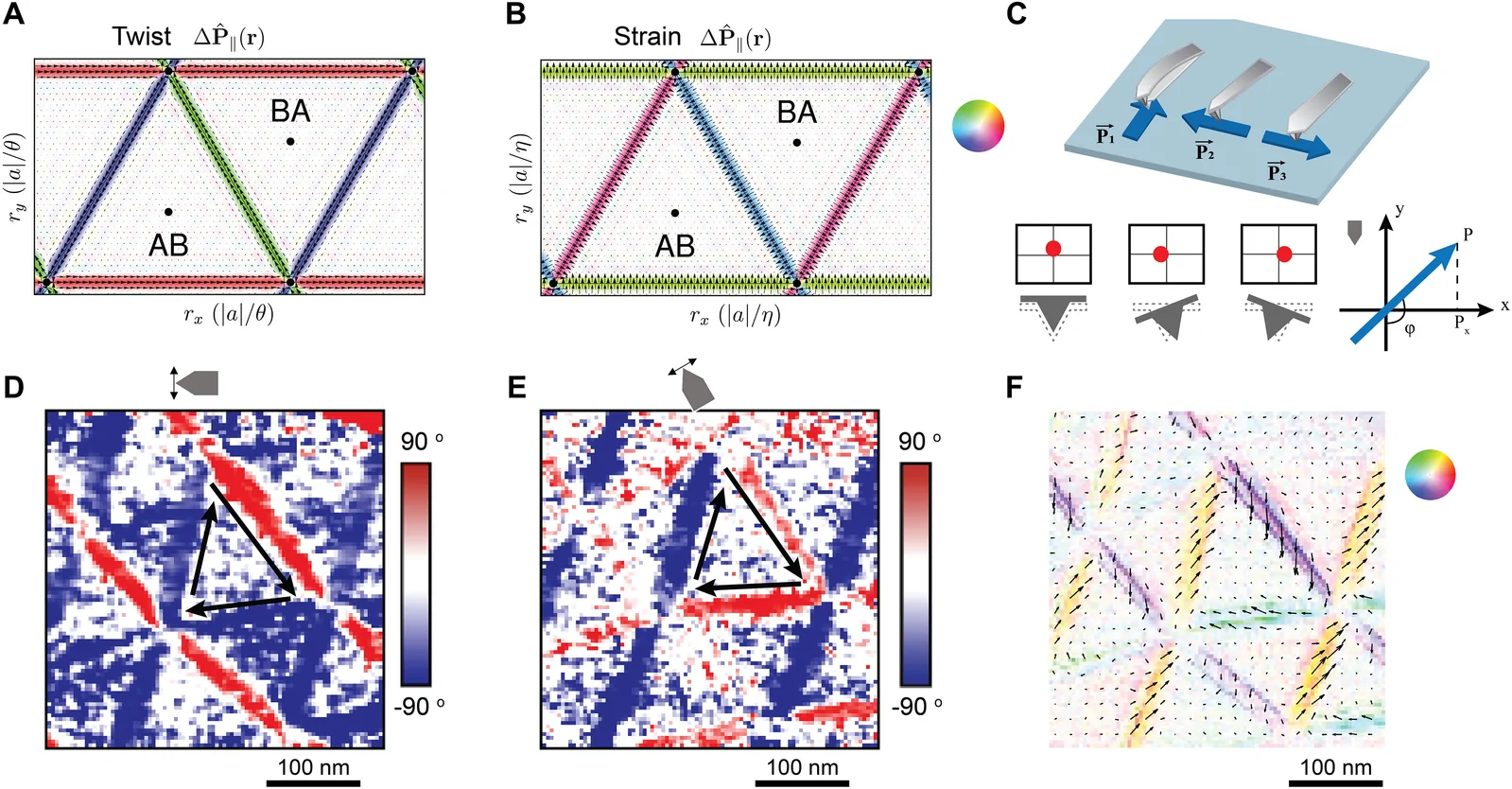
In-plane polarization distribution in marginally twisted bilayer WSe_2_. Theoretically calculated changes in in-plane polarization $P_{∥}\left(r\right)$ for (**A**) twisted and (**B**) biaxially strained (lattice mismatch) WSe_2_ after lattice relaxation. The lattice is scaled by the twist angle θ and lattice mismatch η, respectively. (**C**) The top panel illustrates how in-plane polarization vector $\vec{P}$ (blue arrow) affects sample deformation and cantilever lateral torsion during a PFM measurement. The bottom panel shows deflection on the quadrant photodiode detector of ${\vec{P}}_{1}$ (left), ${\vec{P}}_{2}$ (middle), ${\vec{P}}_{3}$ (right) denoted in the top panel and the intensity of polarization (*P_x_*) as a function of the in-plane polarization at a certain angle (φ) between the cantilever (grey arrow) and the polarization vector (blue arrow). (**D** and **E**) Phase images of lateral PFM measurements, performed on a 0.13° region of bilayer WSe_2_. The grey insets represent the orientation of the cantilever and the double headed arrows represent the scan direction. The one-headed arrows indicate the direction of the in-plane polarization within the domain walls. The direction was determined based on the convention in the bottom panel of (C). (**F**) Angle-Resolved-PFM (AR-PFM) colorized vector field map generated from lateral PFM images in (D and E) as well as several other images.

The theoretically calculated in-plane polarization $P_{∥}\left(r\right)$ in Fig. 2, A and B shows the direction of the in-plane polarization field in the domain walls is different for strained bilayers, i.e. one with a small lattice mismatch between the layers, and for twisted bilayers, i.e. two layers twisted with respect to each other. For twisted bilayers in Fig. 2A, the in-plane polarization is parallel to the domain walls, pointing towards the narrow AA regions, whilst for strained bilayers in Fig. 2B the polarization is perpendicular to the domain walls, pointing towards (away from) the AB (BA) domain centers. To confirm these predictions, we use the lateral deflection to observe whether the phase of the PFM signal changes depending on the domain wall orientation. The illustration in Fig. 2C explains how in-plane polarization ($\vec{P}$) affects sample deformation and thus, affects lateral deflection during a PFM measurement. A single lateral PFM image allows determination of the component of in-plane polarization perpendicular to the tip (*P*_x_) and the polarization sign as shown in the bottom panel of Fig. 2C. To fully map the in-plane polarization vector field, we utilize a well-established angle resolved vector PFM technique (*32*), where PFM measurements taken at different angles between sample and cantilever axis are added together to construct a vector map of the in-plane polarization.

Figure 2, D and E show representative in-plane PFM measurements on a twisted WSe_2_ device with twist angle of 0.13°, at two different orientations of the cantilever axis relative to the sample, as illustrated above each panel. The PFM phase along the domain walls changes as a function of the angle and allows an unambiguous assignment of the polarization directions along the wall, which closes around the triangular domains in a network of clockwise and anticlockwise fashion. Interestingly, this region is close to a bubble, where the MPDs may be affected by both twist and strain due to the bubble. Consequently, a small but measurable phase is detected for the walls that are aligned with the cantilever axis (i.e. Fig. 2D, domain wall at the bottom; Fig. 2E, domain wall at the right), suggesting the presence of a simultaneous polarization component perpendicular to these walls. Figure 2F shows the result of the vector PFM analysis constructed from Fig. 2, D and E as well as several other images (for further details on this method, see section S4). These in-plane vector PFM maps visualize and make the winding of the polarization immediately clear. In this region the shape of the elucidated in-plane polarization field runs slightly diagonal along the domain walls, demonstrating this moiré superlattice results from both twist (Fig. 2A) and strain (Fig. 2B) and establishes in-plane/torsional PFM as an effective and direct method for differentiating between twisted and strained moiré superlattices.

**Fig. 3.**
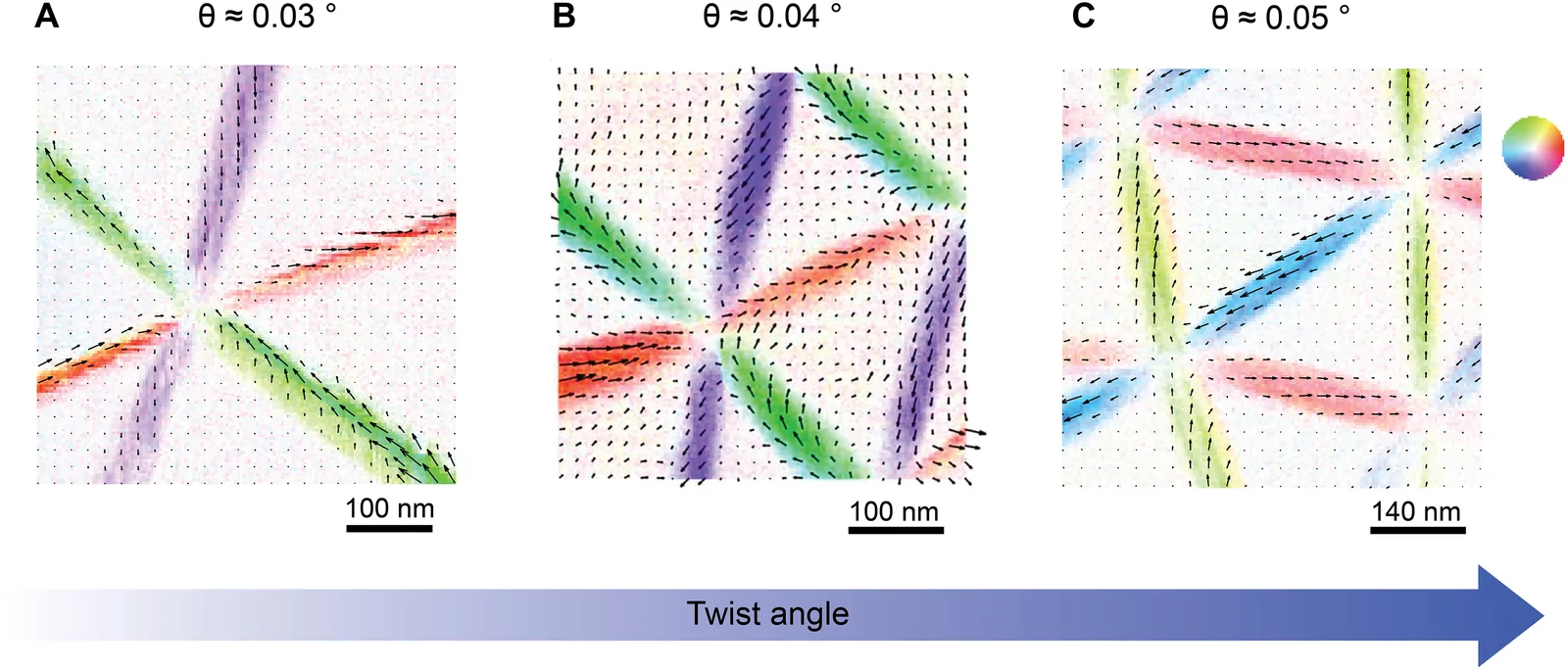
Imaging the angular dependence of in-plane polarization. (**A** to **C**), Angle-resolved-PFM (AR-PFM) colorized vector field maps with varying twist angles generated from lateral PFM images (see section S5).

**Fig. 4.**
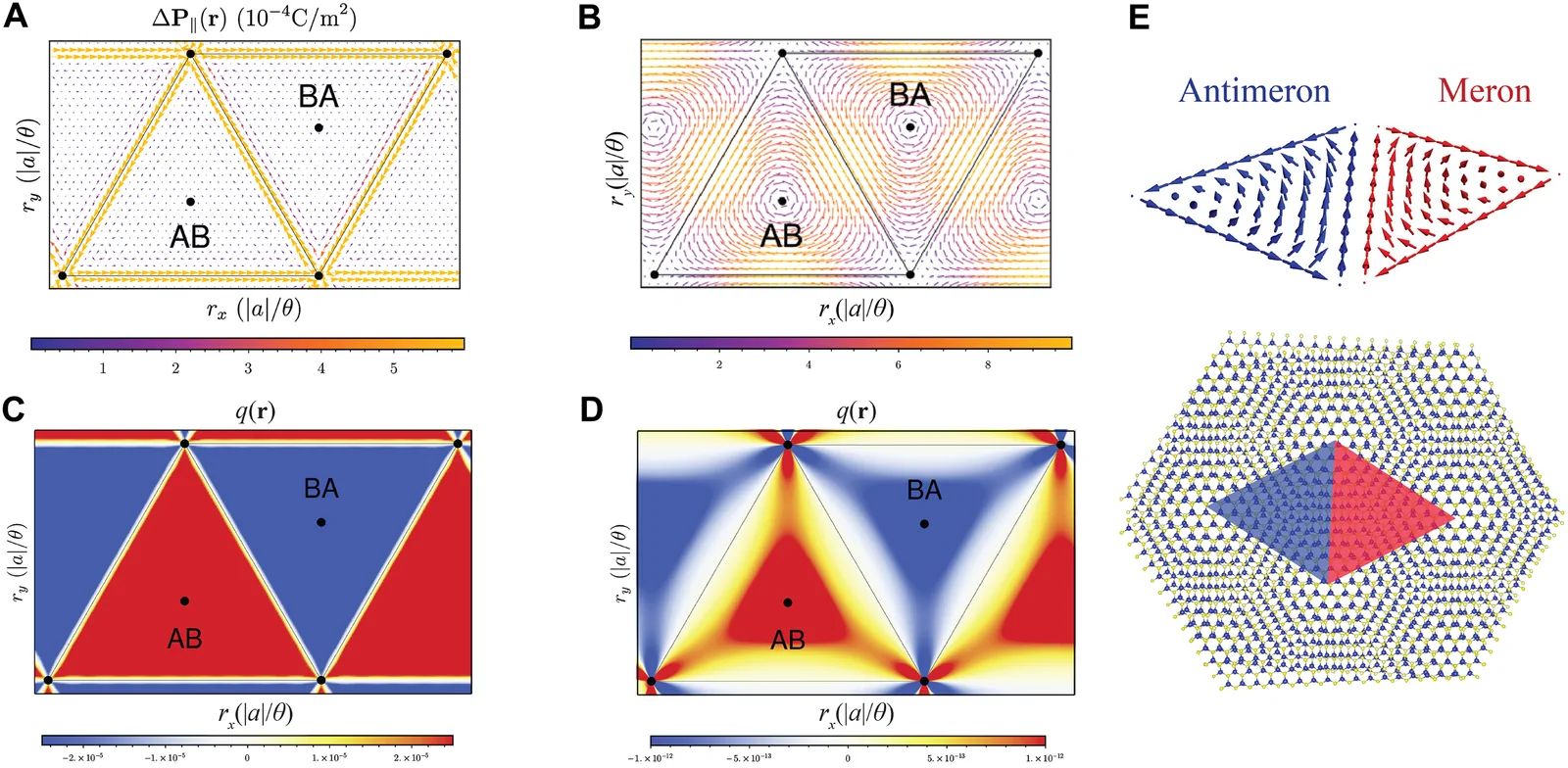
Theoretical calculation of polarization textures in twisted WSe_2_. (**A**) In-plane polarization in a 0.13° twisted bilayer WSe_2_, obtained from DFT calculations. The effects of lattice relaxation on the stacking domains are included. (**B**) In-plane polarization in a 0.13° bilayer WSe_2_, obtained from MD simulations. In-plane polarization is acquired through parameterization facilitated by DFT calculations. (**C** and **D**) Topological charge, i.e. the winding of the total polarization field, from (C) DFT calculations and (D) MD simulations. The polarization is normalized everywhere except for the AA stacking, where both out-of-plane and in-plane components are zero. (**E**) Schematic illustration of a twisted bilayer where the two AB and BA polar domains are highlighted, and the merons and antimerons which form are sketched above.

To gain statistically robust information, full angular dependent PFM measurements were performed on a set of three marginally twisted WSe_2_ devices with twist angles of 0.03°, 0.04° and 0.05°, whose in-plane vector PFM maps are shown in Fig. 3, A to C respectively. The corresponding angular dependent lateral PFM amplitude and phase images are shown in section S5. In these regions (which were scanned away from bubbles and wrinkles) the in-plane polarization is almost entirely parallel to the domain walls, meaning that within these regions of bilayer WSe_2_ the MPD arises entirely due to twist. The angular dependence of the polarization response was further confirmed from lateral PFM measurements as a function of the relative angle (φ) between each domain wall orientation and the cantilever direction. For each domain, the polarization response was quantified by measuring the PFM lateral amplitude after performing a background subtraction and combining it with the sign of the polarization direction determined from the lateral phase. The resulting data points were normalized then plotted as a function of the relative angle φ, in figs. S5 to S7 and then fitted using the function: $y=y_{0}+\text{Asin}\left(\pi \frac{\varphi -\varphi_{c}}{w}\right)$ where φ_c_ is a phase shift, *w* is the period, *A* is the amplitude, and *y*_0_ is the offset. This yielded phase shifts of −1.7°, 2.4° and 0.6° for the 0.03°, 0.04° and 0.05° twisted bilayer WSe_2_ devices respectively, confirming the in-plane polarization is almost entirely parallel to the domain walls. This is in excellent agreement with theoretical predictions (Figs. 2A and 4, A and B) that, after lattice relaxation, the in-plane polarization is confined to the domain walls, as the interior of the domains has nearly uniform AB/BA stacking, and in-plane polarization is prevented by C_3_ symmetry. We note that for these measurements, the in-plane phase difference was much smaller than the ideal 180° but is still sufficient to demonstrate the topological winding (see section S6). The observed much smaller phase difference can arise from a range of factors, including losses associated with tip-sample mechanical coupling, contact stiffness, and other non-idealities, as has been reported in many of the previously published works, including on classical ferroelectric oxides, where the reported phase difference ranges from a few degrees to tens of degrees (*14*, *26*, *33*, *34*). Moreover, our measurements were performed across various PFM tips and scanning conditions (e.g. PFM excitation frequencies and amplitudes, scan angles, etc. Refer to sections S8 and S9), and topological winding of the in-plane polarization vector field is always observed in moiré superlattices, which further attests to the intrinsic nature of the topological winding.

First-principles DFT calculations were performed in order to validate the observation of in-plane polarization in the twisted and strained regions of bilayer WSe_2_ (see Methods), and to verify the topological nature of the MPDs. The out-of-plane and in-plane polarization in bilayer WSe_2_ was calculated as a function of relative stacking between the layers. The out-of-plane polarization has a maximum and minimum at the AB and BA stackings, and is zero for the AA and DW stackings. The in-plane polarization is zero for the AA, AB and BA stackings, and is largest for the DW stacking. In order to accurately describe the polarization field observed in the sample, the notable lattice relaxation which occurs at small angles (*23*) is taken into account (see Methods). The resulting in-plane polarization for bilayer WSe_2_ with a relative twist angle of 0.13° is shown in Fig. 4A. The in-plane polarization is confined to and points parallel to the narrow domain walls, with negligible polarization inside the MPDs, in excellent agreement with the vector maps in Figs. 2F and 3, A to C. Combining the in-plane and out-of-plane (fig. S2), the winding (topological charge) of the total polarization was calculated (Fig. 4C). We note that total polarization is calculated as the dipole moment divided by the volume *V* = *Ad*, where *A* is the in-plane area of the primitive unit cell, and the interlayer separation *d* is taken to be the separation between the metals in each layer. The winding is of opposite sign in the AB and BA MPDs, and integrates to ±½, confirming the topological nature of the experimentally measured polarization in marginally twisted bilayer WSe_2_: a network of Bloch type merons and antimerons (Fig. 4E).

In addition to DFT calculations, large-scale MD calculations were also performed in order to determine the structure of bilayer WSe_2_ twisted at an angle of 0.13° (see Methods). This numerical calculation offers the advantage of encompassing all atoms within the moiré supercell. Starting with two layers of WSe_2_ with a global twist of 0.13°, the system was relaxed in order to determine the equilibrium geometry of the bilayer. The resulting in-plane polarization is shown in Fig. 4B, which also shows polarization confined to the domain walls and circulating around the domain centers. Combining the in-plane and out-of-plane (see fig. S2), the topological charge was calculated (Fig. 4D), which also indicates a network of Bloch type merons and antimerons. We note that, although the structural properties differ between DFT calculations, which predict sharper stacking domains, and more realistic large-scale MD calculations, both methods verify the nature of the in-plane polarization and out-of-plane polarization, as experimentally measured, and confirm the topological nature of the MPDs.

## DISCUSSION

In this work, we demonstrate that in-plane PFM measurements can be used to detect complex polarization textures in moiré superlattices. We have directly confirmed that the MPDs in a twisted TMD have an in-plane texture, and this can be used to directly probe whether MPDs arise due to twist or strain, thus providing a simple and effective method to differentiate between the two components in moiré superlattices. Furthermore, by using angular-dependent in-plane PFM we demonstrate the in-plane polarization in twisted WSe_2_ is parallel to the domain walls, and winds around the AB and BA domain centers, in excellent agreement with two independent theoretical predictions. This confirms the topological nature of the MPDs, i.e. that they form a regular network of merons and antimerons. In contrast to the polar skyrmions typically observed in oxide perovskite superlattices (*35*) and recently in moiré oxide heterostructures (*36*), typically tens of nanometers in thickness, the meron-antimeron network observed here is truly two-dimensional in nature.

The techniques developed in this work represent a method for measuring complex and topological polar structures, which, for the case of bilayers comprised of TMDs or hBN, are difficult to determine using other methods typically used to detect topological polar structures in oxide perovskites (*19*, *22*, *23*, *35*), as the polarization is electronic and are difficult to determine solely by measuring the individual displacements of the atoms. We anticipate these techniques will play an important role in further exploration and understanding of the connections between twist and topology in other twisted 2D systems, across a wide span of twist angles and layer configurations. Finally, non-trivial real-space topology in twisted van der Waals heterostructures will open pathways in engineered nano-devices, where the manipulation of polar topology can be realised via electric gating (*16*), mechanical deformation (*37*) or by engineering substrates and/or additional 2D layers with tailored strain, doping or twisting.

## MATERIALS AND METHODS

### Sample fabrication

We used blue tape to exfoliate hexagonal boron nitride (*h*BN) onto a conductive silicon wafer, then selected a flat flake with suitable thickness under the optical microscope. We prepared WSe_2_ monolayers on polydimethylsiloxane (PDMS) substrates using the same method. The monolayer nature was confirmed by both optical contrast and photoluminescence spectroscopy. Their orientation was determined by second harmonic generation spectroscopy. The WSe_2_ monolayers were transferred onto the *h*BN one by one by dry transfer method and aligned to each other to obtain marginally twisted bilayer WSe_2_. After each transfer, the sample was cleaned by diisopropylamine and annealed in an argon environment at 100°C for 1 hour. Finally, the sample was annealed in ultra-high vacuum at 270°C for 8 hours before measurements.

### Piezoresponse force microscopy

PFM measurements were performed on a commercial scanning probe microscope, i.e. Bruker Dimension Icon, at room temperature with a Nanoscope 6 controller. We used conductive platinum-iridium coated Bruker probes with tip end radius of 25 nm and spring constant of around 3 N m^−1^. AC bias was applied through the tip, and induced sample deformation whose amplitude and phase represent the magnitude of the piezoelectric coefficient and the polarization direction of the response, was detected respectively. For both vertical and lateral PFM, AC bias voltages were set in the range of 2 V – 3 V and frequencies were around 250–280 kHz. In our measurement, the tip frequency was set near the contact resonance frequency. Contact resonance frequency is where the tip and the sample are in resonance as shown in fig. S9. Here, we have a very good signal-to-noise ratio but mechanical response and piezoelectric response will be mixed. To avoid these issues measurements for all samples (i.e. twist angle of 0.03°, 0.04°, 0.05° and 0.13°) were performed at totally off-resonance frequencies as well (see section S8).

### First-principles calculations

First-principles density functional theory (DFT) calculations were performed using the SIESTA (*38*) code, using PSML norm-conserving pseudopotentials, obtained from Pseudo-Dojo (*39*). SIESTA employs a basis set of numerical atomic orbitals (NAOs), and double-ζ polarized (DZP) orbitals were used for all calculations. A Monkhorst-Pack **k**-point grid (*40*) of 12 × 12 × 1 was used for the initial geometry relaxations, and a mesh of 18x18x1 was used to calculate the polarization. Calculations were converged until the relative changes in the hamiltonian and density matrix were both less than 10^−6^. The C09 (*41*) van der Waals exchange-correlation functional was used to treat the long-range interactions between the layers. A dipole correction was employed in the vacuum region to prevent dipole-dipole interactions between periodic images.

The top layer was translated along the unit cell diagonal over the bottom layer, which was held fixed. At each point a geometry relaxation was performed to obtain the equilibrium layer separation, while keeping the in-plane lattice vectors fixed. The out-of-plane and in-plane polarization were then obtained by calculating the Berry phases of the Bloch states. The data were fitted to Fourier expansions which respect the 𝓒_3_ rotation symmetry t-WSe_2_ (*16*).

### Lattice relaxation

Lattice relaxation calculations were performed following the methodology in Refs (*10*, *11*)., for t-WSe2 twisted at an angle of θ = 0.13° with respect to the ideal rhombohedral stacking (perfectly aligned layers). The total energy of t-WSe_2_ is given by

$$V_{\text{tot}}=\int V_{\text{tot}}\left[x+u\left(x\right)\right]dx$$

$$V_{\text{tot}}\left(x\right)=V_{\text{stack}}(x)+V_{\text{elastic}}\left(x\right)$$

where $V_{\text{tot}}\left(x\right)$is the total energy density as a function of relative stacking **x** between the layers, and **u(x)** is a displacement field which describes the relaxation of the bilayer from its rigid twisted configuration. The integration is performed in “configuration space” (*24*), in terms of the relative stackings between the layers, all of which are contained in a single primitive cell of WSe_2_. The total energy density is given as a sum of two independent terms. The stacking energy, $V_{\text{stack}}\left(x\right)$

$$V_{\text{stack}}\left(x\right)=\sum_{n}\;{V^{e}}_{n}\;{{ɸ}^{e}}_{n}(x)+{V^{o}}_{n}\;{{ɸ}^{o}}_{n}\left(x\right)$$

describes the vdW or cohesive energy between the layers. It is written as a Fourier expansion using even and odd 𝓒_3_ symmetric basis functions ɸ^e/o^_n_

$${{ɸ}^{e}}_{1}=\text{cos}\left(\text{2πx}\right)+\text{cos}\left(\text{2πy}\right)+\text{cos}\text{[2π}\left(x+y\right)\text{]}$$

$${{ɸ}^{e}}_{2}=\text{cos}\text{[2π}\left(x-y\right)\text{]}+\text{cos}\text{[2π}\left(\text{2x}+y\right)\text{]}+\text{cos}\text{[2π}\left(x+\text{2y}\right)\text{]}$$

$${{ɸ}^{e}}_{3}=\text{cos}\left(\text{4πx}\right)+\text{cos}\left(\text{4πy}\right)+\text{cos}\text{[4π}\left(x+y\right)\text{]}$$

$${{ɸ}^{o}}_{1}=\text{sin}\left(\text{2πx}\right)+\text{sin}\left(\text{2πy}\right)-\text{sin}\text{[2π}\left(x+y\right)\text{]}$$

$${{ɸ}^{o}}_{2}=\text{sin}\text{[2π}\left(y-x\right)\text{]}+\text{sin}\text{[2π}\left(\text{2x}+y\right)\text{]}-\text{sin}\text{[2π}\left(x+\text{2y}\right)\text{]}$$

$${{ɸ}^{o}}_{3}=\text{sin}\left(\text{4πx}\right)+\text{sin}\left(\text{4πy}\right)-\text{sin}\text{[4π}\left(x+y\right)\text{]}$$

where x and y are fractions of the primitive lattice vectors of WSe_2_. The elastic energy,

$$\begin{array}{c} V_{\text{elastic}}\left(x\right)=\\ \frac{\theta^{2}}{2}\left\{B{\left(\partial_{x}u_{y}-\partial_{y}u_{x}\right)}^{2}+\mu [{\left(\partial_{x}u_{y}+\partial_{y}u_{x}\right)}^{2}+{\left(\partial_{x}u_{x}-\partial_{y}u_{y}\right)}^{2}]\right\} \end{array}$$

describes the elastic penalty of deforming the layers where Band μ are the bulk and shear modulus respectively. The total energy V_tot_ was minimized to obtain the displacement field **u**(**x**) for fixed values of θ.

The out-of-plane polarization, which is odd with respect to stacking, is given by

$$P_{⟂}\left(x\right)=\sum_{i}\;{P^{⟂}}_{n}\;{{ɸ}^{o}}_{n}\left(x\right)$$

The in-plane polarization is even with respect to stacking, and thus the vector basis functions can be given by ∇_**x**_ɸ^o^_n_(**x**)

$$P_{∥}\left(x\right)=\sum_{i}\;{P^{∥}}_{n}\;{\text{∇}}_{x}{{ɸ}^{o}}_{n}\left(x\right)$$

The coefficients P^⟂^_n_ and P^**∥**^_n_ are obtained by fitting the polarization obtained from DFT calculations to 𝓒_3_-symmetric odd and even scalar fields and vector fields, respectively.

The resulting polarization field including the effects of lattice relaxation **P**[**x + u**(**x**)] for WSe_2_ with a twist angle of 0.13° is shown in Fig. 4, A and B.

The winding of the polarization

$$q\left(x\right)=P\left(x\right)\cdot \left[\partial_{x}P\left(x\right)\;\times \;\partial_{y}P\left(x\right)\right]$$

where **x** = (x,y) and **P**(**x**) is normalized, is shown in Fig. 4C. The winding was calculated following the methodology in Ref. (*16*), on a fine real space grid, offset from the AA stacking by half a grid spacing, the only point where the normalized polarization is not well-defined. Integrating the winding in the AB and BA domains yields a total winding of Q_AB_ = +½ (meron) and Q_BA_ = -½ (meron), respectively.

### Molecular Dynamics calculations

In addition to DFT calculations, lattice relaxations were also performed using molecular dynamics (MD) simulations. We utilized MD simulations employing the Large-scale Atomic Molecular Massively Parallel Simulator (LAMMPS) (*42*) and classical interatomic force field models for atomic relaxation.

For the case of twisted WSe_2_, we applied the KC potential for interlayer interactions and the SW potential for intralayer intralayer interactions with SW/mod style (*43*, *44*). Lattice relaxation calculations were conducted for a commensurate twist angle θ = 0.13°, involving approximately 1 million atoms. Utilizing the relaxed atomic positions, the in-plane displacement is computed between the bottom and top layers. Subsequently, the determination of the out-of-plane and in-plane of polarization, as well as the topological charge is achieved via parameterization, facilitated by the DFT calculations. The quantitative charge values differ between MD and DFT calculations due to the utilization of distinct grid sizes in the analysis. Nevertheless, qualitatively, the AB and BA configurations exhibit opposite winding behaviour and converge to ±½.

## Supplementary Materials

### This PDF file includes:

Sections S1 to S11 Supplementary Text Figs. S1 to S12
