# Supplementary material for: Imaging topological polar structures in marginally twisted 2D semiconductors
Source: Sci Adv. 2026 Sep 11;12(37):eaed8555. doi: 10.1126/sciadv.aed8555 (PMC13564965; doi:10.1126/sciadv.aed8555)
Supplement: Supplementary file 1 [file sciadv.aed8555_sm.pdf]

Science Advances
AAAS

Supplementary Materials for

Imaging topological polar structures in marginally twisted 2D semiconductors

Thi-Hai-Yen Vu *et al.*

Corresponding author: Daniel Bennett, daniel.bennett@ntu.edu.sg; Pankaj Sharma, pankaj.sharma@flinders.edu.au; Michael S. Fuhrer, michael.fuhrer@monash.edu; Mark T. Edmonds, mark.edmonds@monash.edu

Sci. Adv. **12**, eaed8555 (2026)
DOI: 10.1126/sciadv.aed8555

This PDF file includes:

Sections S1 to S11
Supplementary Text
Figs. S1 to S12

S1. Optical image and second harmonic generation of twisted bilayer WSe_2 device

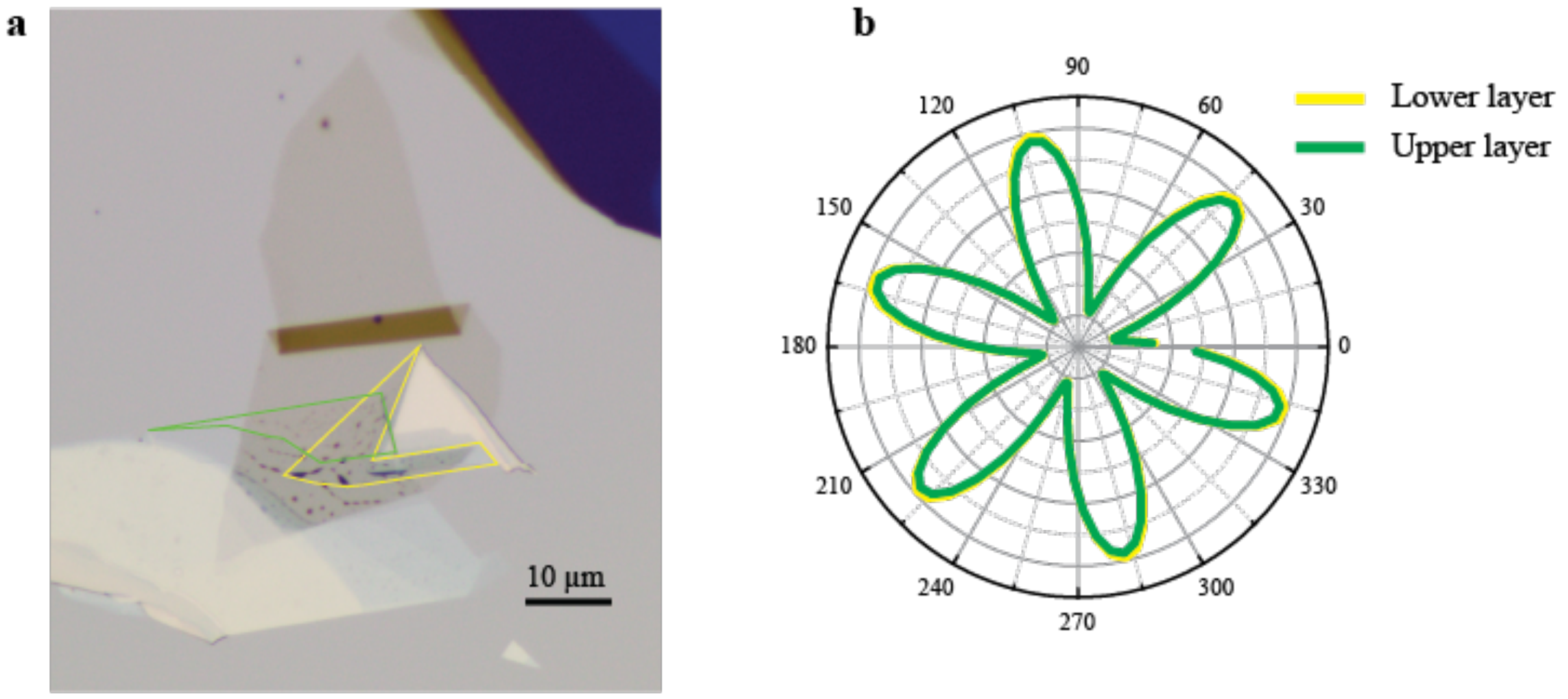

Figure S1. a, Optical image of stacked WSe_2. Upper and lower monolayers WSe_2 are highlighted by green and yellow colours. **b,** Fitted second harmonic generation (SHG) spectroscopy of upper and lower monolayer WSe_2 after stacking showing the near zero twist angle.

S2. AFM topography, PFM amplitude and phase images.

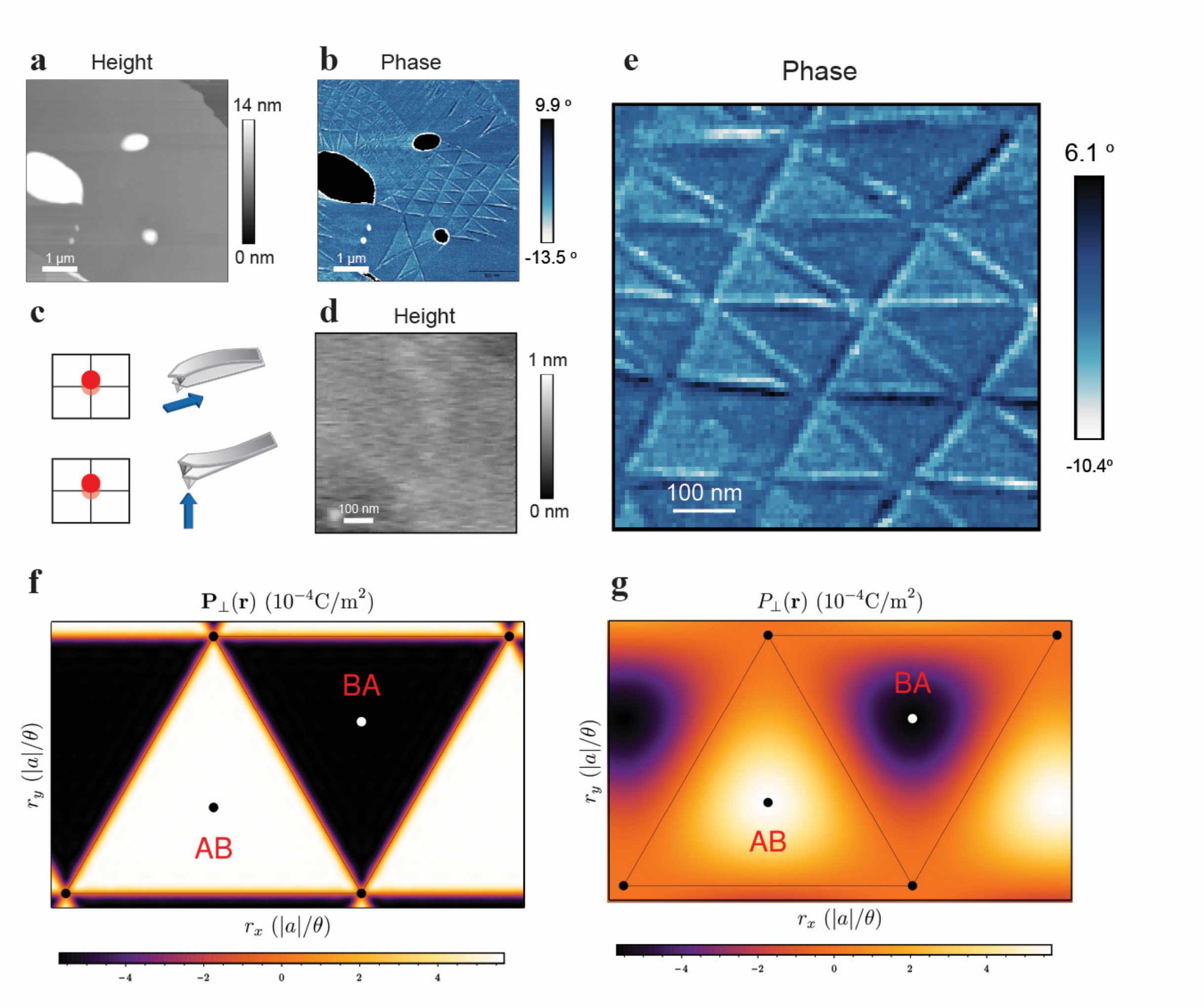

Figure S2. a, b, Comparison of topography and phase images obtained of the region shown in Fig. 1(d) of the main manuscript. **c,** Illustration of vertical torsion and deflection resulting from out-of-plane polarization during a vertical PFM measurement. **d, e** Topography and corresponding vertical phase images of a zoomed in region of a,b. There is a strong vertical PFM signal near the DWs because they have large strain gradients which result in a polar response through flexoelectricity (*26*). Noted that the in-plane flexoelectric response is negligible (*26*), see Section S3 for additional details. This couples to piezoelectric stresses, resulting in two strong lines at the DWs in the vertical PFM signals. **f, g,** Out-of-plane polarization in bilayer WSe_2 with a twist angle of 0.13°, obtained from **f** DFT calculations and **g** MD calculations. The effects of lattice relaxation on the stacking domains are included.

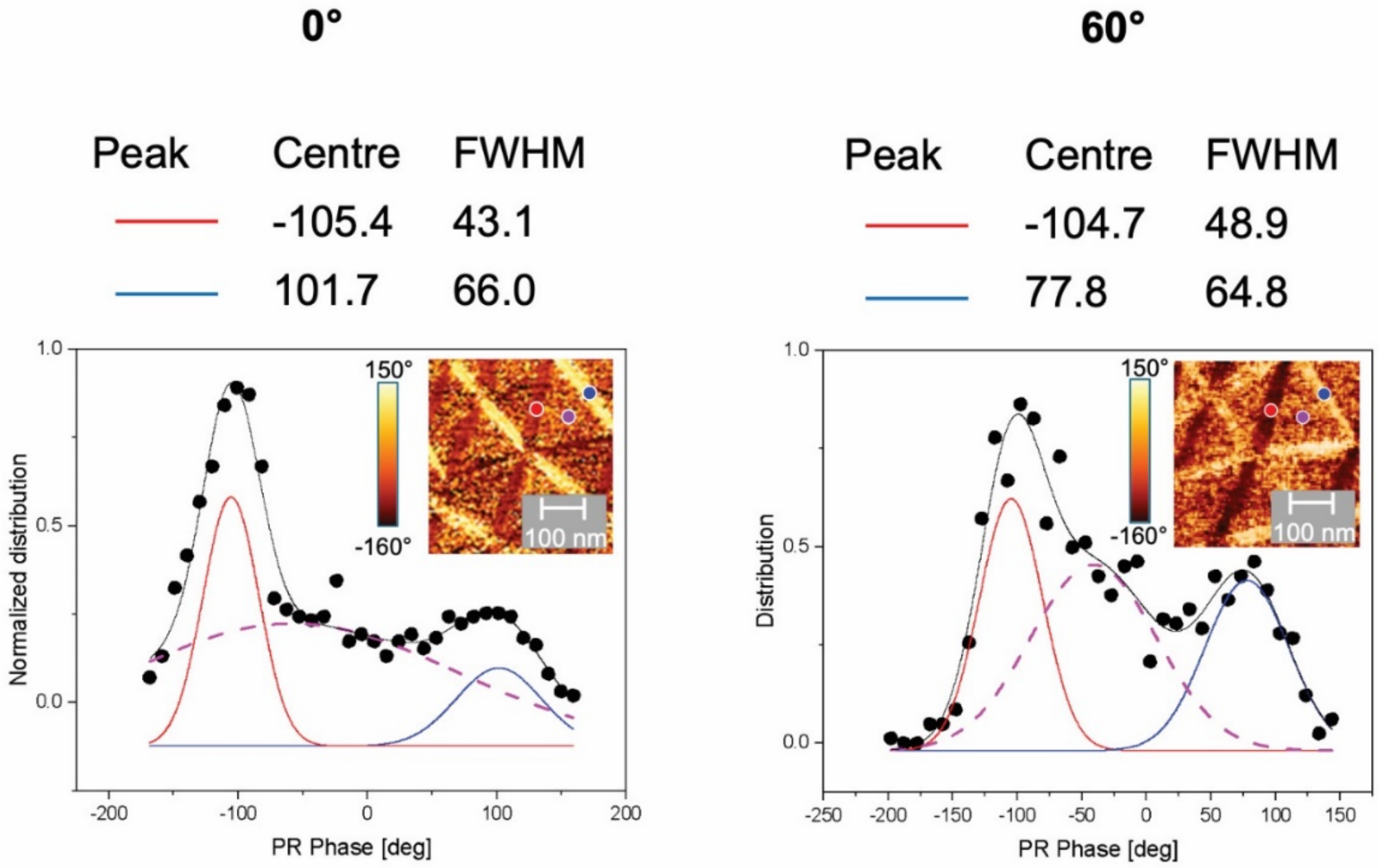

Figure S3. Histogram analysis of lateral PFM phase images taken at 0º and 60º showing near 180º phase switch.

S3. Discussion of in-plane flexoelectricity

The flexoelectric response in twisted bilayers has been considered in detail in Figure 4h of McGilly *et al.* (*26*), where they show that the flexoelectric response is localized to the domain walls, where the strain gradient is largest. Furthermore, they show that the most significant flexoelectric response is an out-of-plane polarization arising from a bending mode, with the other (in-plane) responses being negligible.

The in-plane flexoelectric response in twisted bilayers proposed in McGilly *et al.*: Counter-flowing polarization along the outside of the domain walls, with zero polarization exactly in the middle of the domain wall. We do not see this in our measurements: the in-plane polarization flows in the same direction within each direction. Therefore, we can rule out in-plane flexoelectricity in our measurements.

S4. Generating angle-resolved-PFM (AR-PFM) images

To generate the in-plane angle-resolved-PFM (AR-PFM) images or vector map, the lateral PFM signals taken at different angles (i.e. between sample and cantilever long axis) were determined from their respective amplitude and phase channels. This was achieved by first centring the phase data around 0 degrees and taking the sine of the phase angle multiplied by the amplitude.

Image alignment was conducted using the phase channels as they were clearest. A set of vertices visible in each scan and near to the region of interest were chosen and identified for each scan angle. One scan (the zero-degree angle scan) was designated to be the reference. For the remaining scans, an affine transformation was calculated using the reference vertices, optimised to minimise least squares error.

Once aligned, the x- and y- components of the vector field could be calculated. This was done by projecting the in-phase component along the scan direction for each scan angle. Then the resultant map was created by summing all the contributions and rescaling if necessary.

The resultant vector field was visualised by a hue-saturation map and a superimposed quiver map. For the hue-saturation map, the hue of a pixel corresponds to the angle of the vector and the saturation corresponds to the magnitude of the vector.

S5.= Angular Dependent PFM for 0.03°, 0.04° and 0.05° marginally twisted WSe_2 bilayers

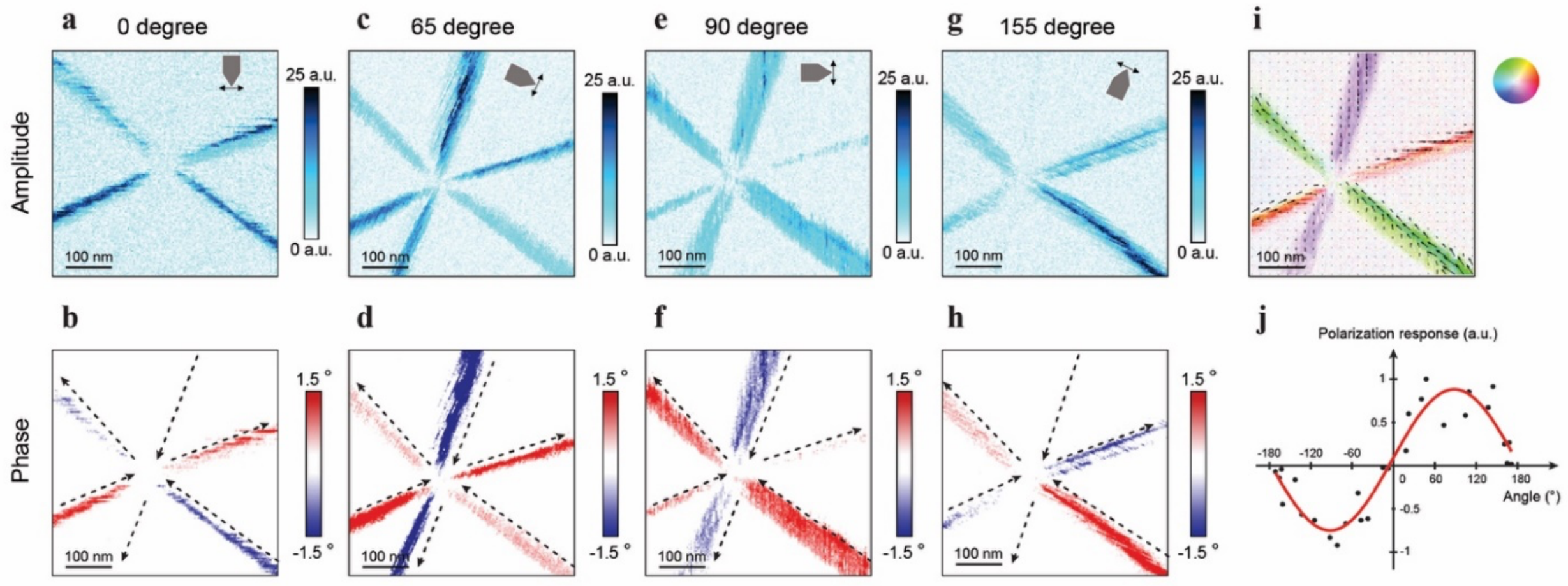

Figure S4. Angular dependent PFM of marginally twisted (0.03º) bilayer WSe_2**. a-h**, In-plane amplitude and phase images of lateral PFM of the same moiré polar domain when the relative angle between sample and cantilever Φ is **a,b** 0°, **c,d** 65°, **e,f** 90° **g,h** 155°. **i,** Corresponding Angle-Resolved-PFM (AR-PFM) vector map. **j,** Normalized polarization response as a function of in-plane polarization angle φ. The phase offset is -1.7±4.1º.

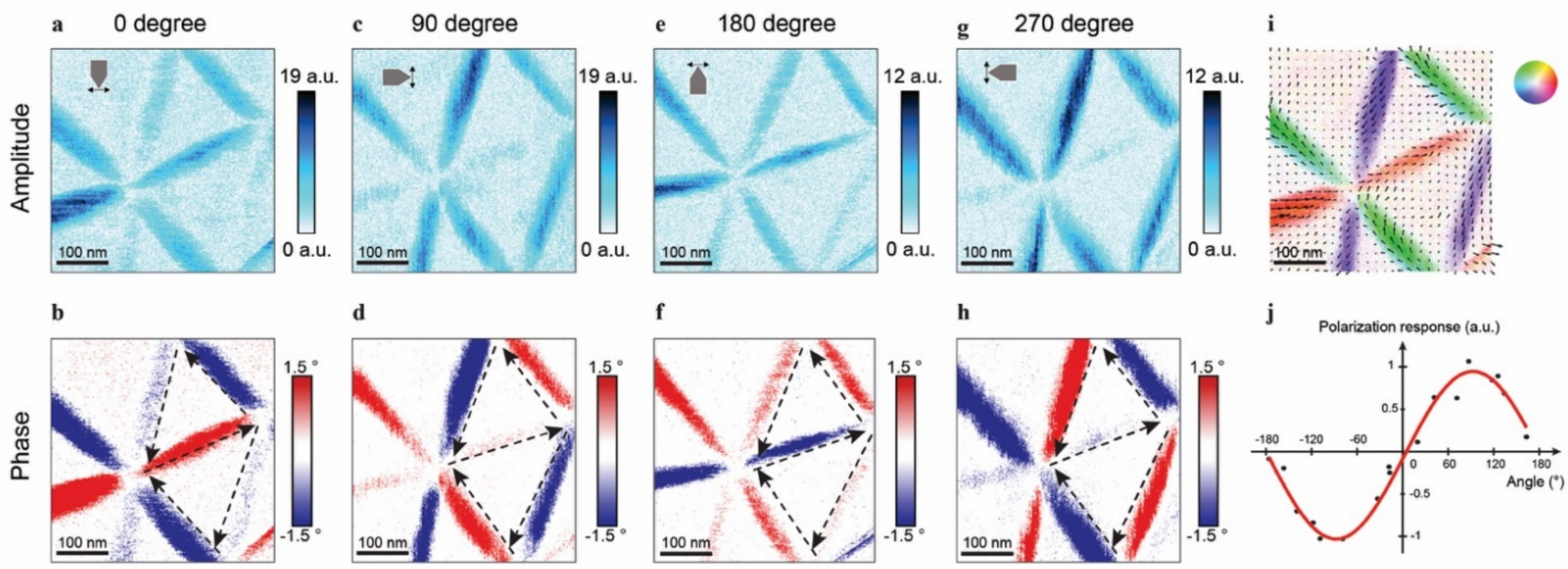

Figure S5. Angular dependent PFM of marginally twisted (0.04º) bilayer WSe_2**. a-h**, In-plane amplitude and phase images of lateral PFM of the same moiré polar domain when the relative angle between sample and cantilever Φ is **a,b** 0°, **c,d** 90°, **e,f** 180° **g,h** 270°. **i,** Corresponding Angle-Resolved-PFM (AR-PFM) vector map. **j,** Normalized polarization response as a function of in-plane polarization angle φ. The phase offset is 2.4±2.8º.

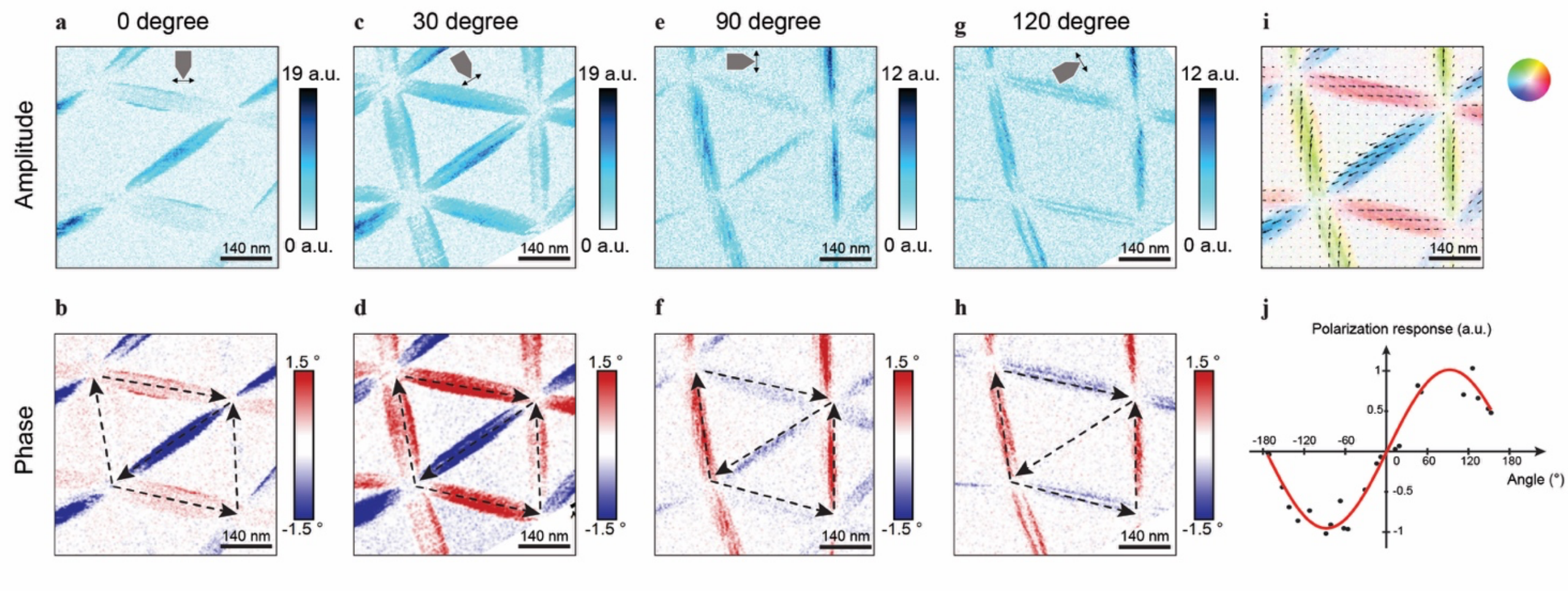

Figure S6. Angular dependent PFM of marginally twisted (0.05º) bilayer WSe_2**. a-h**, In-plane amplitude and phase images of lateral PFM of the same moiré polar domain when the relative angle between sample and cantilever Φ is **a,b** 0°, **c,d** 30°, **e,f** 90° **g,h** 120°. **i,** Corresponding Angle-Resolved-PFM (AR-PFM) vector map. **j,** Normalized polarization response as a function of the in-plane polarization angle φ. The phase offset is 0.6±2.5º.

S6.= Comparison of 180° and small phase shifts with corresponding vector maps.

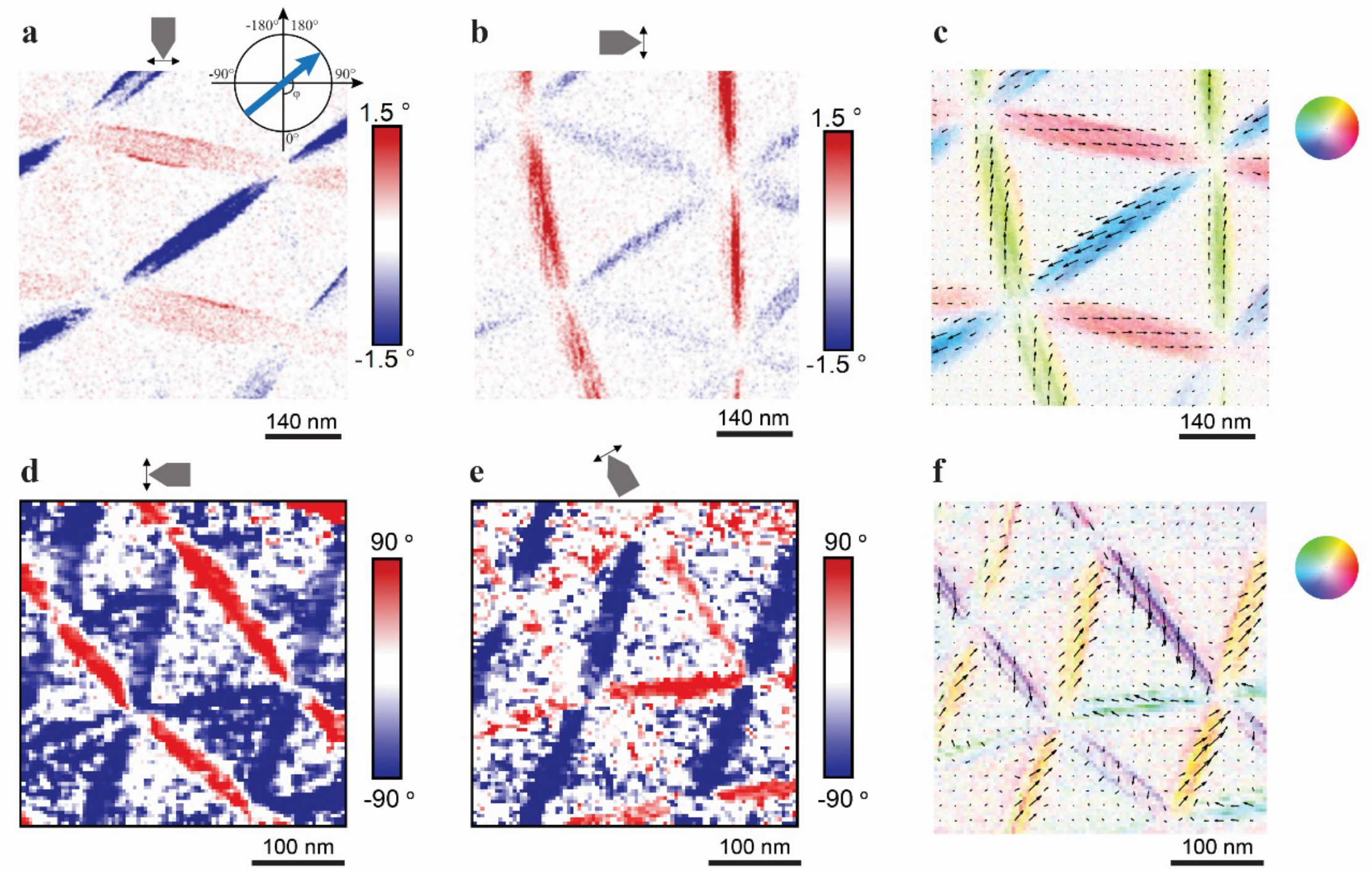

Figure S7. a-b, In-plane phase images of lateral PFM of the moiré polar domain when the relative angle between sample and cantilever Φ is **a** 0° **b** 90°. **c,** AR-PFM colorized vector field map generated from lateral PFM images in **a–b**. **d-e**, In-plane phase images of lateral PFM of the moiré polar domain when the relative angle between sample and cantilever Φ is **d** 0° **e** 60°. **f,** AR-PFM colorized vector field map generated from lateral PFM images in d–**e.**

S7. PFM contact resonance frequency curve

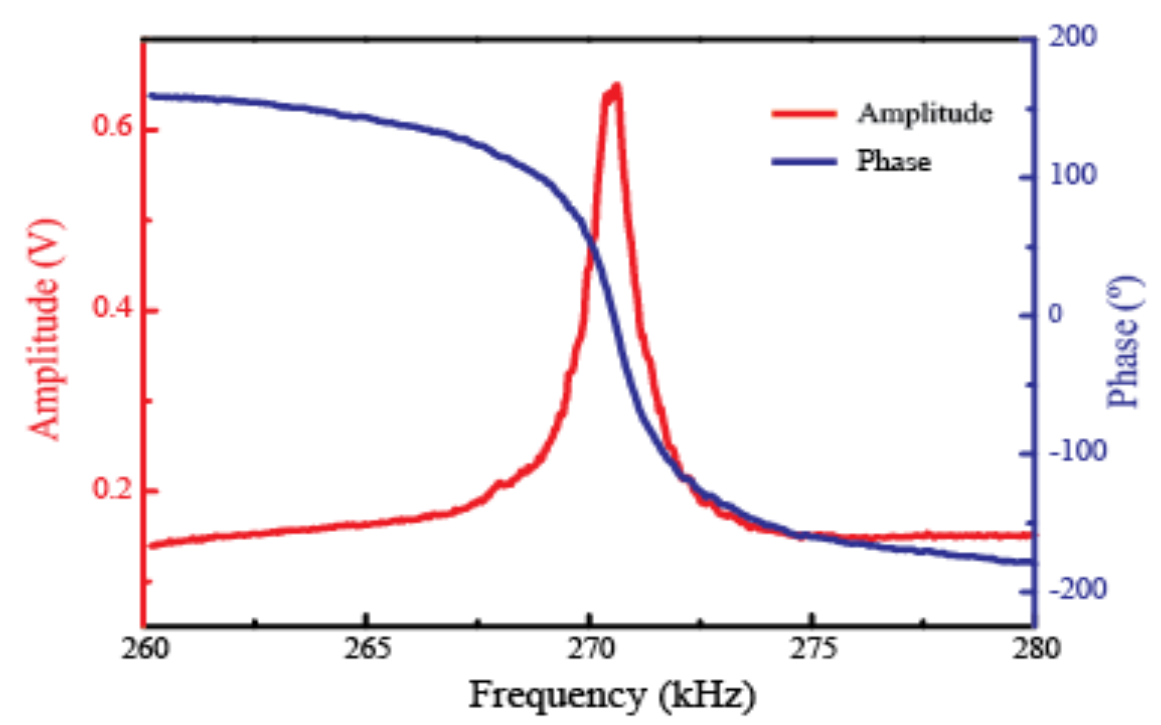

Figure S8. Contact resonance frequency in PFM.

S8. Comparing measurement near-resonance and off-resonance frequencies

To achieve more stable signals during repeated piezoresponse mapping and to minimize cross-talk between in-plane and out-of-plane responses – issues that are amplified in resonance-enhanced modes such as dual AC resonance tracking – we decided to use off-resonance conditions. Importantly, the results obtained at both near-resonance and off-resonance frequencies showed no notable differences in the measured domain structures as shown in Fig. S9 below. All features across the three samples remain the same, and consistent with winding of the polarization.

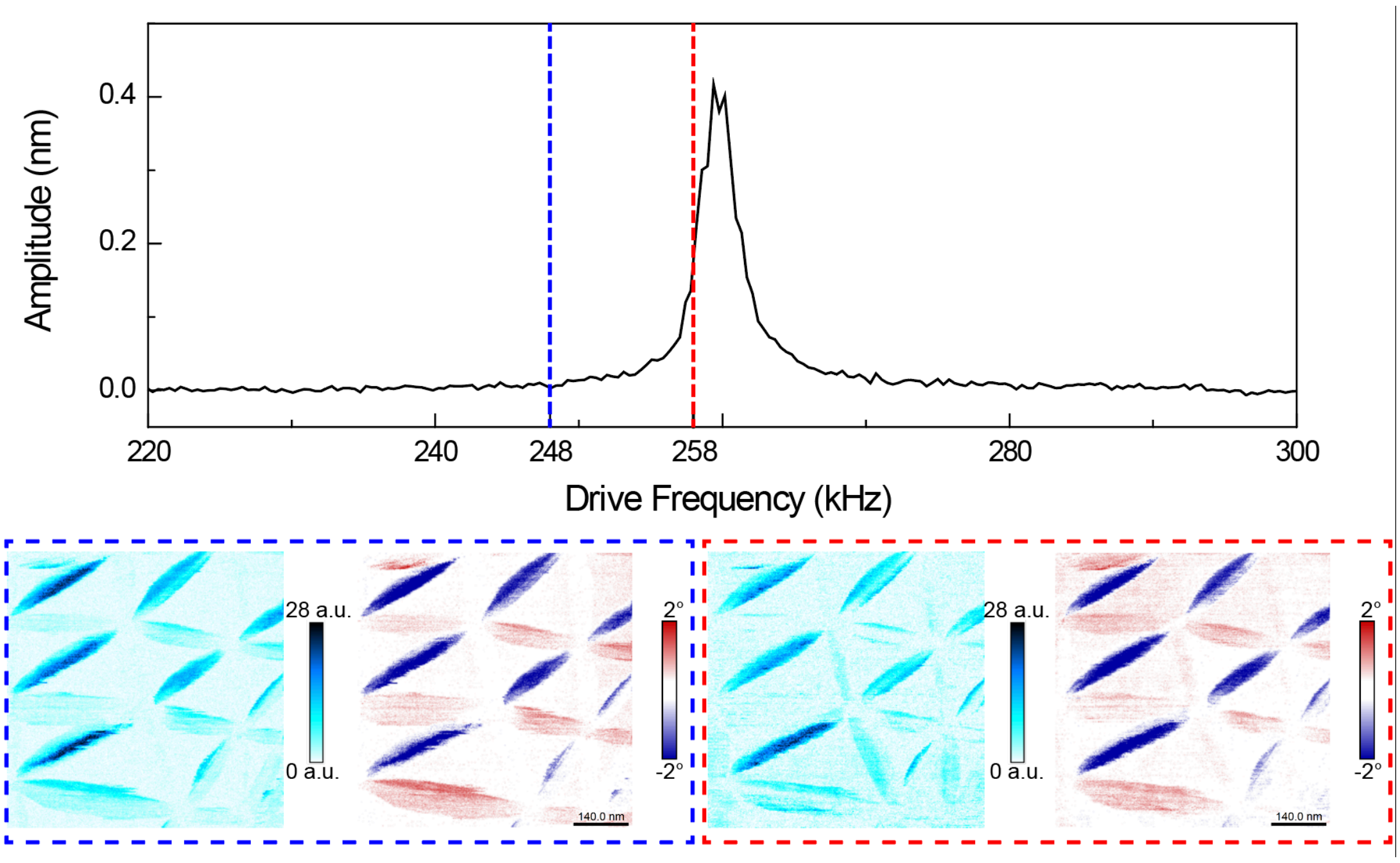

Figure S9. The top graph shows the contact resonance frequency spectrum in PFM, where the near-resonance and off-resonance drive frequencies are marked with red and blue dashed lines, respectively. The bottom images display the corresponding PFM measurements. Images in the red box represent near-resonance lateral amplitude and phase, images in the blue box show off-resonance lateral amplitude and phase.

S9. Measurements with tip radius of 5 nm and 25 nm

We performed additional measurements using a PFM tip with a smaller tip radius: 5 nm compared with the regular tip which has a 25 nm radius. After performing measurements with higher resolution, we observed a narrowing of the domain walls, but the winding of the polarization was consistently observed.

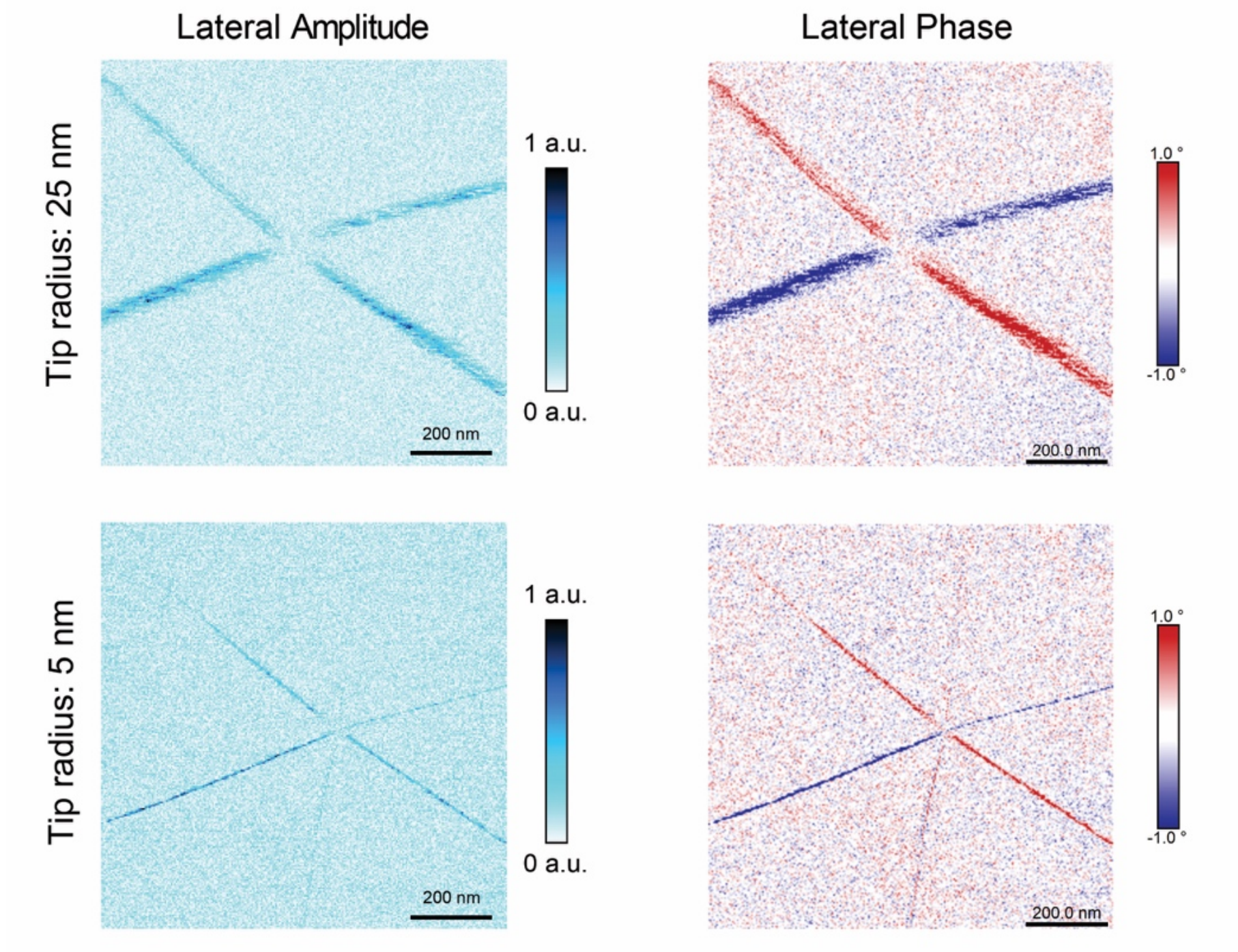

Figure S10. Lateral PFM amplitude and phase images were acquired for the same region using a tip with a 5 nm and 25 nm radius.

S10. Molecular dynamics simulations of temperature dependence of twisted WSe_2 bilayers.

To investigate the temperature dependence of the winding, we performed molecular dynamics simulations to investigate the stability of twisted WSe_2 bilayers. The WSe_2 bilayers were first relaxed using standard minimization. Once the structure was relaxed, molecular dynamics simulations were performed in the NVT ensemble (constant number of particles, volume, and temperature). The simulations were performed at two distinct temperatures: 100 K and 300 K. Fig. S11 shows the fluctuations of the average interlayer separation as a function of time. The results show that in both temperature regimes, the system equilibrates over time, with the interlayer distance fluctuating around a stable equilibrium value. At both temperatures, the system initially exhibits significant fluctuations that diminish as the system approaches equilibrium. This behaviour is characteristic of systems transitioning from a non-equilibrium to an equilibrium state, where thermal energy is dissipated, and the system stabilizes at its minimum energy configuration. These results clearly demonstrate that at finite temperatures, the WSe_2 bilayer undergoes thermal fluctuations around an equilibrium interlayer distance. The extent of these fluctuations appears to reduce as the system equilibrates, suggesting that the layers are dynamically stable, with the interlayer distance consistently fluctuating around a stable value after the equilibrium is reached. This analysis offers valuable insights into the thermal stability of WSe_2 layers, indicating that they remain in a stable configuration at both 100 K and 300 K, despite the inherent thermal fluctuations at higher temperatures.

The shape of the polarization field depends on the local stackings in the twisted supercell. Because the stackings do not dramatically change at room temperature, only fluctuating slightly about equilibrium values, the winding of the polarization and its topological nature persists at room temperature. This is further supported by our measurements of the winding, performed at room temperature, which are in excellent agreement with theoretical predictions (zero temperature).

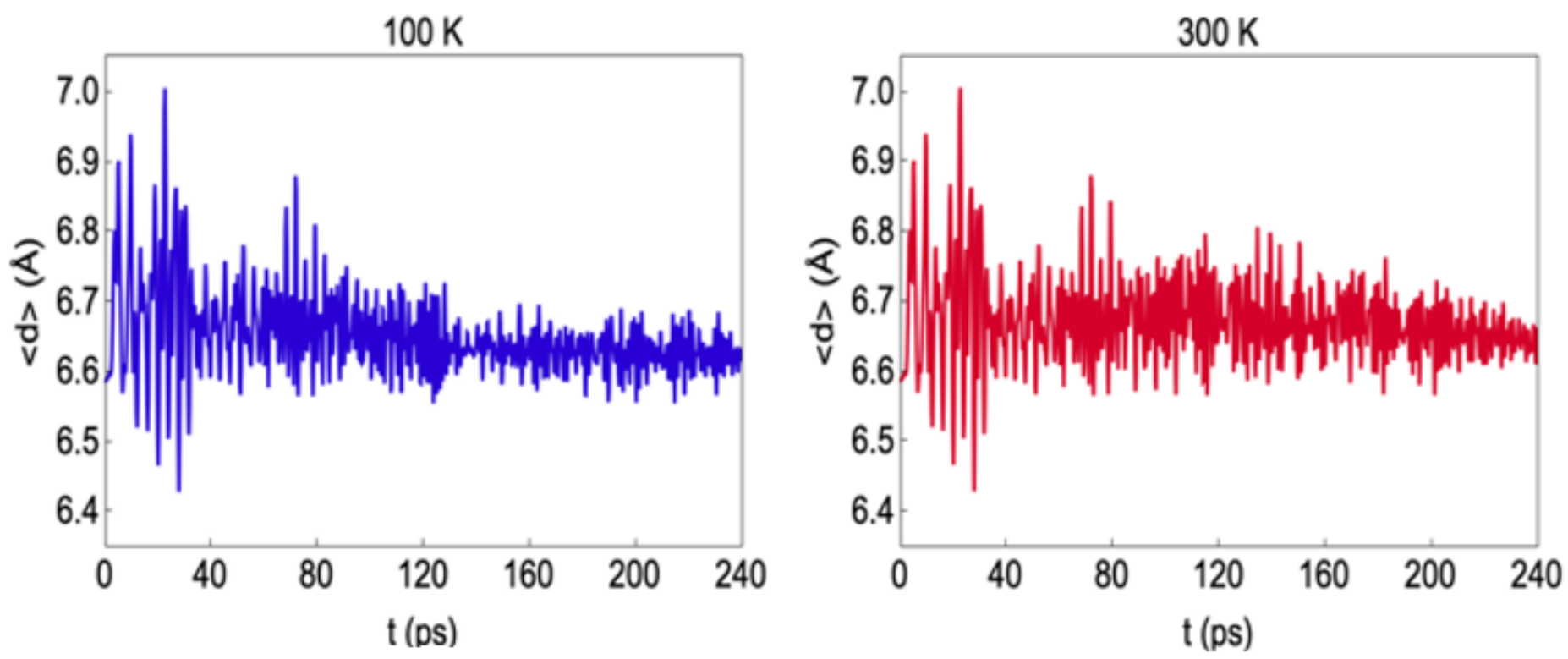

Figure S11. Fluctuations in the average interlayer distance <d> as a function of time t for temperatures T=100 K and T=300 K as obtained from LAMMPS molecular dynamics simulations. The plot shows the temporal evolution of <d> at each temperature, highlighting the differences in thermal motion and system behaviour at lower and higher temperatures.

S11. Periodically-poled lithium niobite standard sample measurement at off-resonance frequency

To verify there was no cross-talk from out-of-plane polarization to lateral PFM, we performed measurements on the Bruker standard PFM sample, which consists of periodically poled lithium niobate with pure out-of-plane polarization. Using the same off-resonance frequency settings as for our twisted sample, we observed a 180° phase difference in vertical PFM, consistent with out-of-plane polarization switching. Meanwhile, the lateral PFM showed minimal signal, indicating negligible contribution of the vertical domains to the lateral signal at domain walls under our experimental setup. This minimal signal is significantly smaller than the lateral PFM signals observed in our twisted bilayers.

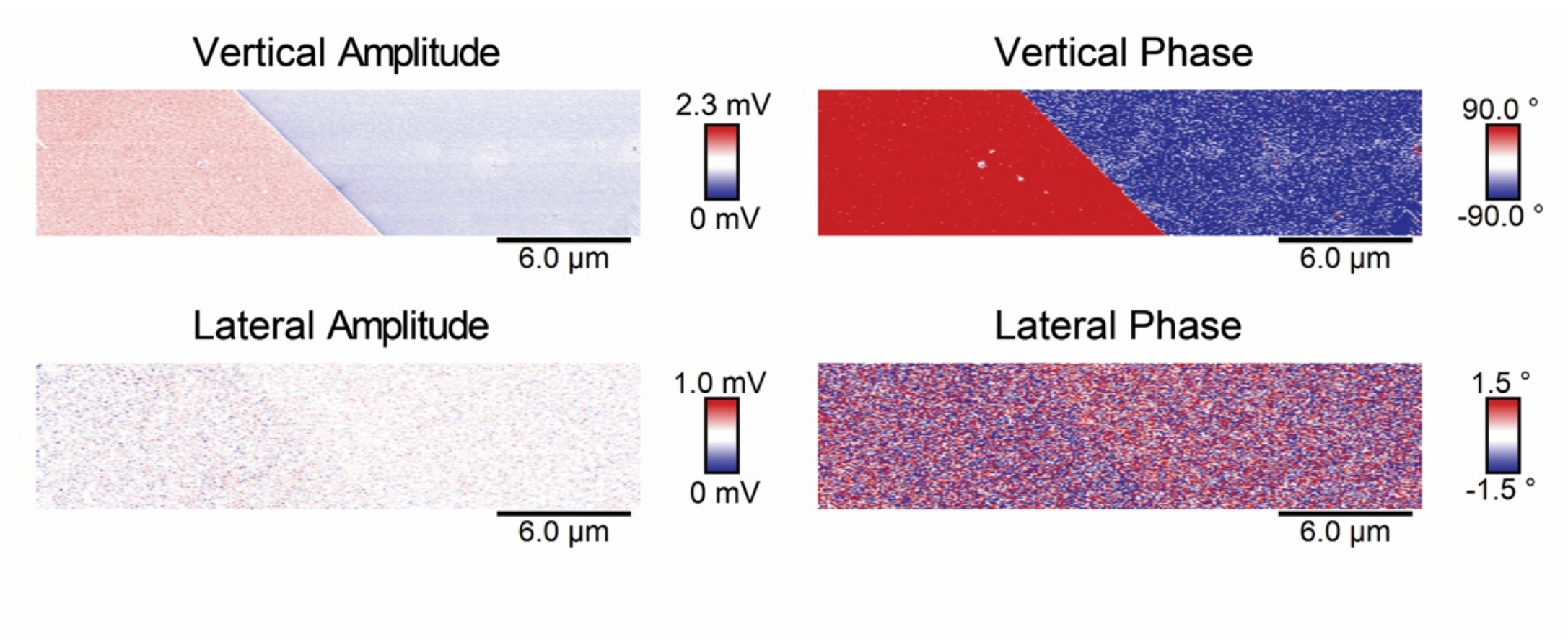

Figure S12. PFM images of periodically-poled lithium niobate standard sample.
